# Lifestyle factors associated with benign multiple sclerosis

**DOI:** 10.1136/jnnp-2024-335464

**Published:** 2025-02-13

**Authors:** Jie Guo, Tomas Olsson, Jan Hillert, Lars Alfredsson, Anna Karin Hedström

**Affiliations:** 1 China Agricultural University, Beijing, Beijing, China; 2 Department of Clinical Neuroscience, Karolinska Institute, Stockholm, Sweden; 3 Karolinska Institute Institute of Environmental Medicine, Stockholm, Stockholm, Sweden

**Keywords:** MULTIPLE SCLEROSIS, Prognosis

## Abstract

**Background:**

Benign multiple sclerosis (MS), characterised by minimal disability despite long disease duration, remains poorly understood in terms of its determinants and prognostic implications. While lifestyle factors have been implicated in modifying disease progression, their role in distinguishing benign and non-benign MS remains unclear.

**Methods:**

We conducted a comparative analysis of patients with benign (n=2040) and non-benign MS (n=4283) using data from Swedish nationwide case-control studies with long-term follow-up. Logistic regression models were used to analyse associations between a history of infectious mononucleosis (IM) and lifestyle factors (smoking, body mass index, fish consumption and sun exposure habits) and the likelihood of benign MS. Additionally, Cox regression was used to follow patients with benign MS from the 15-year mark onward, identifying factors associated with the transition to non-benign MS over time.

**Results:**

The odds of having benign MS were reduced in association with a history of IM (OR 0.54, 95% CI 0.45 to 0.65), adolescent overweight and obesity (OR 0.69, 95% CI 0.56 to 0.85 and 0.46, 95% CI 0.32 to 0.66, respectively) and infrequent fish consumption (OR 0.72, 95% CI 0.60 to 0.88). Similar associations were observed for the risk of transitioning from benign to non-benign MS over time.

**Conclusions:**

A history of IM and modifiable lifestyle factors significantly influence the probability of a benign disease course in MS. These findings underscore the potential for targeted lifestyle interventions to improve MS outcomes. Further research is needed to elucidate the mechanisms by which a past IM infection can continue to influence MS progression long after the initial infection.

**WHAT IS ALREADY KNOWN ON THIS TOPIC**

- Lifestyle factors, such as smoking, obesity and dietary habits, have been shown to influence multiple sclerosis (MS) progression, but their role in distinguishing benign from non-benign MS has not been clearly defined.

**WHAT THIS STUDY ADDS**

- This study provides new evidence that a history of infectious mononucleosis and modifiable lifestyle factors, including adolescent overweight/obesity and fish consumption, are significantly associated with the likelihood of benign MS.

**HOW THIS STUDY MIGHT AFFECT RESEARCH, PRACTICE OR POLICY**

- Our findings highlight the importance of addressing modifiable risk factors, such as obesity and dietary habits, as part of MS management strategies to potentially improve long-term outcomes. These results may influence clinical decision-making by identifying patients at higher risk of non-benign MS who could benefit from more aggressive monitoring or interventions.

## Introduction

 The concept of benign multiple sclerosis (MS) is controversial. Defined retrospectively, benign MS describes a subset of patients who exhibit minimal disability after many years of living with the disease, typically characterised by an Expanded Disability Status Scale (EDSS) score of 3.0 or less after 10–15 years.^1 2^ Although the definition of benign MS is based on the EDSS score which mainly reflects physical disability, studies investigating the long-term trajectories of MS courses have found that patients with benign MS also have a lower rate of cognitive decline and higher health-related quality of life.^3 4^ Despite its seemingly favourable prognosis, the term benign is controversial due to the unpredictable nature of MS and the potential for progression even after many years of stability.^5 6^

One of the primary challenges with the concept of benign MS is its limited clinical value, as it is based on past disease courses and provides little guidance for future management. Currently, it is impossible to accurately identify which patients will follow a benign course at the time of diagnosis. However, understanding the factors that influence the course of MS is of paramount importance. Clinical variables that have been associated with benign MS are female sex, young age at onset, few relapses during the first 2 years from onset and complete recovery from the first relapse.^3 4^ The main MS risk HLA genes have been found not to be associated with a benign or a non-benign course.^4^

In this study, we aimed to compare patients with benign and non-benign MS using data from nationwide population-based case-control studies with long-term follow-up. By examining environmental exposures and lifestyle habits, we seek to identify potential factors that may contribute to the differences in disease course between benign and non-benign MS.

## Methods

The Epidemiologic Investigation of Multiple Sclerosis (EIMS) and Genes and Environment and Multiple Sclerosis (GEMS) are Swedish nationwide population-based case-control studies, comprising the general population aged 16–70 years. Between April 2005 and December 2019, EIMS recruited 3567 newly diagnosed patients with MS from hospital-based neurology units and privately run clinics across Sweden. GEMS identified prevalent cases, distinct from those in EIMS, from the Swedish MS registry^7^ between November 2009 and November 2011 (n=6148). The response rate among cases was 92% in EIMS and 82% in GEMS. All patients were diagnosed by local neurologists according to the McDonald criteria.^8 9^ All participants provided information on environmental exposures and lifestyle habits completing a standardised questionnaire. More details on the study design and methods are provided elsewhere.^10^

Benign MS was defined as having an EDSS score of 3 or lower after at least 15 years of disease duration. Non-benign MS was defined as having reached an EDSS score exceeding 3 before 15 years of disease duration. Of the 9715 patients, we excluded those who were not followed-up with EDSS scores in the Swedish MS registry (n=626) and those with an EDSS of 3 or less within 15 years (n=1941) since it was not possible to classify them as either benign or non-benign MS according to our study criteria. We also excluded those with progressive-onset MS (n=676) and those with uncertain disease phenotype (n=149). Our study comprised 2040 benign and 4283 non-benign patients.

### Definition of exposures

The questions from the EIMS and GEMS questionnaires are provided in Online supplemental file 1.

**Education level:** Self-reported education level, validated by information retrieved from the National Board of Health and Welfare, was dichotomised at the threshold of obtaining a university degree, as this distinction reflects a meaningful divide in socioeconomic status, cognitive reserve and access to resources that are relevant to MS outcomes.

**Infectious mononucleosis:** Infectious mononucleosis (IM) was dichotomised into yes or no.

**Smoking:** Smoking was dichotomised into ever or never smoker at disease onset, despite available pack-year data, to avoid misclassification due to unknown smoking cessation after disease onset.

**Body mass index:** Body mass index (BMI) was calculated by dividing self-reported weight in kilograms by height in metres squared. BMI at age 20 years was categorised into clinically relevant thresholds (<25, 25–30, >30 kg/m^2^) for interpretability and to ensure robust statistical analysis, despite the availability of exact BMI measures.

**Fish consumption:** Participants reported their average frequency of oily fish consumption at study inclusion. Additionally, GEMS reported fish consumption at age 20 years. For this study, we focused on fish consumption at the time of inclusion in both cohorts. Oily fish species were defined as those with a fat content of more than 3%, such as herring, mackerel, tuna, salmon and trout. This threshold has been commonly used in dietary research to distinguish oily fish from lean fish. Responses were recorded on a 4-point scale: never/seldom, 1–3 times/months, weekly or daily. Due to the small number of participants reporting daily fish consumption, the weekly and daily categories were combined. Low fish consumption was defined as never/seldom consuming fish, moderate consumption as 1–3 times/month and high consumption as weekly or more frequent consumption.

**Sun exposure:** Sun exposure was assessed using three questions regarding ultraviolet radiation exposure, with responses recorded on a 4-point scale. The responses were summed to create a composite index ranging from 3 to 12, representing overall sun exposure. The median value of the index 6 was used to dichotomise sun exposure into low or high.

### Statistical analysis

Categorical variables were summarised using frequency and percentage. Continuous variables were summarised using mean and SD. Comparisons between benign and non-benign MS groups were performed using t-tests for continuous variables and chi-square tests for categorical variables.

Logistic regression models were used to calculate the OR with 95% CIs of having benign MS for each exposure. The analyses were performed based on the overall sample and in a subanalysis limited to those of Nordic origin. Additionally, we conducted the analysis limited to untreated patients and those treated only with low/moderate-efficacy therapies during the first 15 years of the disease course and to those on high efficacy therapies. This approach aimed to assess the robustness of our findings regarding lifestyle factors and the probability of a benign disease course while controlling for potential confounding by the indication for more intensive therapies. Adjustments were made for potential confounders, including age at disease onset, sex, ancestry, disease-modifying therapy and other lifestyle habits. We accounted for the influence of treatment by calculating the proportion of the first 15 years of disease duration spent on low/moderate-efficacy and high-efficacy therapies. Relapses during the first 2 years and recovery from the first relapse were not included as confounding variables, as they are closely linked to the definition of benign MS and reflect the disease course itself rather than independent risk factors.

In a subsequent analysis, we used Cox regression to follow patients classified as benign MS at 15 years and examined the probability of transitioning to non-benign MS over time.

### Sensitivity analyses on benign MS definitions

We conducted a sensitivity analysis by modifying the definition of benign MS to an EDSS score of 3 or less after 10 years of disease duration. Additionally, we tested a stricter definition of benign MS, defined as an EDSS score of less than 3 after 15 years of disease duration. All analyses were conducted using the Statistical Analysis System V.9.4.

## Results

Patients with benign MS were younger at disease onset compared with those with non-benign MS (p<0.0001) and more often of Nordic origin (p<0.0001). Significant differences were observed between benign and non-benign MS groups concerning past IM, BMI in young adulthood and fish consumption habits. Characteristics of benign and non-benign patients with MS are presented in table 1. A lower proportion of the patients in the benign group received high-efficacy therapies compared with the non-benign group (22% vs 47%), while the use of low-efficacy therapies was more comparable (40% vs 41%). Additionally, a higher proportion of patients in the benign group remained untreated during the first 15 years of disease duration compared with the non-benign group (37% vs 12%).

**Table 1:** Characteristics of benign and non-benign patients with MS

	Total	Benign	Non-benign	
Total	6323	2040	4283	
Age at disease onset (SD)	32.2 (10.1)	29.3 (8.6)	33.5 (10.4)	<0.0001
Age at diagnosis (SD)	37.2 (10.8)	37.5 (11.2)	37.1 (10.7)	0.11
Male, n (%)	1677 (26.5)	553 (27.1)	1124 (26.2)	0.47
Nordic ancestry, n (%)	5540 (87.6)	1872 (91.8)	3668 (85.6)	<0.0001
University examination, n (%)	1132 (17.9)	342 (16.8)	790 (18.5)	0.10
Infectious mononucleosis, n (%)	843 (13.3)	190 (9.3)	653 (15.3)	<0.0001
Ever smoker at disease onset, n (%)	3343 (52.9)	1071 (52.5)	2272 (53.1)	0.64
BMI at age 20 years, kg/m^2^ (SD) BMI<25 kg/m^2^, n (%) Overweight, n (%) Obesity, n (%)	22.0 (3.7) 5501 (87.0) 618 (9.8) 204 (3.2)	21.5 (3.4) 1846 (90.5) 155 (7.6) 39 (1.9)	22.2 (3.8) 3655 (85.3) 463 (10.8) 165 (3.9)	<0.0001
Fish consumption on 4-grade scale (SD) Never or seldom, n (%) 1–3 times/month, n (%) Weekly, n (%)	2.2 (0.6) 728 (11.8) 3519 (57.2) 1910 (31.0)	2.3 (0.6) 182 (9.1) 1080 (54.2) 729 (36.6)	2.2 (0.6) 546 (13.1) 2439 (58.6) 1181 (28.4)	<0.0001
Low sun exposure at disease onset, n (%)	1974 (31.2)	631 (30.9)	1343 (31.4)	0.73
Disease-modifying treatment within the first 15 years of disease duration, n (%)	5051 (80.0)	1282 (62.8)	3769 (88.0)	<0.0001
Time between disease onset and treatment start (SD)	3.5 (3.8)	6.7 (5.4)	3.4 (3.6)	<0.0001

BMI, body mass index; MS, multiple sclerosis.

The OR of having benign MS was 0.54 (95% CI 0.45 to 0.65) among those with a history of IM compared with those who had not. Overweight and obesity in young adulthood were associated with lower probability of benign MS (OR 0.69, 95% CI 0.56 to 0.85 and 0.46, 95% CI 0.32 to 0.66, respectively), with a trend indicating reduced probability of benign MS with increasing BMI (p for trend<0.0001). Compared with consuming oily fish 1–3 times/month, individuals who never or seldom consumed fish had a lower probability of benign MS (OR 0.72, 95% CI 0.60 to 0.88), while those with weekly consumption had a higher probability of benign disease (OR 1.44, 95% CI 1.28 to 1.64). There was a clear trend showing an increased probability of benign MS with higher fish consumption (p for trend<0.0001). These findings remained statistically significant after Bonferroni correction for multiple comparisons. Smoking status and sun exposure at disease onset were not significantly associated with benign MS (table 2). Our findings remained similar when we limited the analysis to participants of Nordic ancestry (online supplemental etable 1). The significant associations of past IM, obesity in young adulthood and fish consumption with benign MS remained consistent when we performed the analysis in groups based on treatment regimens (online supplemental etable 2).

In a subsequent analysis, we followed those classified as benign MS at 15 years using Cox regression to examine the probability of transitioning to non-benign MS over time. In the multivariable analysis, transitioning to non-benign MS was associated with disease onset after age 40 years (HR 1.24, 95% CI 1.08 to 1.42), a history of IM (HR 1.43, 95% CI 1.15 to 1.76), overweight/obesity in young adulthood (OR 1.33, 95% CI 1.05 to 1.68) and weekly fish consumption (OR 0.83, 95% CI 0.72 to 0.95). All associations remained statistically significant after applying Bonferroni correction for multiple comparisons, apart from the association with overweight/obesity (table 3).

**Table 2:** Factors associated with achieving benign MS at 15 years

Characteristic		Exp BMS/non-BMS	OR (95% CI)*	OR (95% CI)†	P value‡
Age at disease onset	<30 years 30–40 years >40 years	1189/1870 630/1336 221/1077	1.0 (reference) 0.74 (0.66 to 0.84) 0.32 (0.27 to 0.38)	1.0 (reference) 0.70(0.61 to 0.79) 0.24 (0.20 to 0.28)	<0.0006 <0.0006
Sex	Female Male	1487/3159 553/1124	1.0 (reference) 1.06 (0.93 to 1.28)	1.0 (reference) 1.15 (1.01 to 1.31)	0.20
Ancestry	Nordic Non-Nordic	1872/3668 168/615	1.0 (reference) 0.55 (0.45 to 0.64)	1.0 (reference) 0.52 (0.43 to 0.63)	<0.0006
University examination	No Yes	1698/3493 342/790	1.0 (reference) 0.89 (0.78 to 1.02)	1.0 (reference) 0.89 (0.77 to 1.04)	0.93
Infectious mononucleosis	No Yes	1850/3630 190/653	1.0 (reference) 0.57 (0.48 to 0.67)	1.0 (reference) 0.54 (0.45 to 0.65)	<0.0006
Smoking	No Yes	969/2011 1071/2272	1.0 (reference) 0.97 (0.88 to 1.08)	1.0 (reference) 1.02 (0.92 to 1.16)	1
BMI at age 20 years	< 25 kg/m^2^ Overweight Obesity	1846/3655 155/463 39/165	1.0 (reference) 0.67 (0.55 to 0.81) 0.47 (0.33 to 0.67)	1.0 (reference) 0.69 (0.56 to 0.85) 0.46 (0.32 to 0.66)	0.002 <0.0006
Fish consumption	Never or seldom 1–3 times/month Weekly	182/546 1080/2439 729/1181	0.76 (0.63 to 0.90) 1.0 (reference) 1.40 (1.25 to 1.57)	0.72 (0.60 to 0.88) 1.0 (reference) 1.44 (1.28 to 1.64)	0.005 <0.0006
Sun exposure	Median or higher Below median	1409/2940 631/1343	1.0 (reference) 0.98 (0.88 to 1.10)	1.0 (reference) 0.99 (0.88 to 1.12)	1

The multi-adjusted models account for all other exposures as well as age at disease onset, sex, ancestry and treatment use during the first 15 years.

* Crude model.

† Multivariate model.

‡ P value adjusted for multiple testing using the Bonferroni method.

BMI, body mass index; BMS, benign multiple sclerosis.

**Table 3:** Factors associated with transitioning from benign MS at 15 years to non-benign MS over time

Characteristic		N	Years (SD)	Outcome (%)	HR (95% CI)*	HR (95% CI)†	P value^‡^
Age at disease onset	≤40 years >40 years	1189 851	17.2 (12.0) 12.7 (8.1)	473 (40) 372 (44)	1.0 (reference) 1.18 (0.98 to 1.41)	1.0 (reference) 1.24 (1.08 to 1.42)	0.009
Sex	Female Male	1487 553	15.3 (10.9) 15.3 (10.6)	626 (42) 219 (40)	1.0 (reference) 0.90 (0.74 to 1.10)	1.0 (reference) 0.92 (0.79 to 1.08)	1
Ancestry	Nordic Non-Nordic	1872 168	15.4 (10.9) 14.4 (10.0)	770 (41) 75 (45)	1.0 (reference) 0.55 (0.45 to 0.64)	1.0 (reference) 1.07 (0.84 to 1.36)	1
University examination	No Yes	1698 342	15.3 (10.9) 15.4 (10.4)	700 (41) 145 (42)	1.0 (reference) 1.05 (0.86 to 1.33)	1.0 (reference) 1.02 (0.85 to 1.22)	1
Infectious mononucleosis	No Yes	1850 190	15.5 (10.8) 13.4 (10.2)	746 (40) 99 (52)	1.0 (reference) 1.61 (1.19 to 2.17)	1.0 (reference) 1.43 (1.15 to 1.76)	0.004
Overweight/obesity at age 20 years	No Yes	1883 157	15.4 (10.8) 13.9 (10.6)	765 (41) 80 (51)	1.0 (reference) 1.52 (1.10 to 2.10)	1.0 (reference) 1.33 (1.05 to 1.68)	0.07
Fish consumption	Less than weakly Weakly	1311 729	14.6 (10.2) 16.9 (11.6)	568 (43) 277 (38)	1.0 (reference) 0.80 (0.67 to 0.97)	1.0 (reference) 0.83 (0.72 to 0.95)	0.04

The multi-adjusted models account for all other exposures as well as age at disease onset, sex, ancestry and treatment use.

* Crude Cox model.

† Multivariate model.

‡ P-value adjusted for multiple testing using the Bonferroni method.

MS, multiple sclerosis.

### Sensitivity analysis

The significant associations between IM and lifestyle factors with the likelihood of a benign disease course were robust across various definitions of benign MS. Notably, when applying the more stringent definition of benign MS, defined as having an EDSS score of less than 3 after 15 years of disease duration, the associations also became slightly more pronounced. For instance, the probability of benign MS associated with past IM shifted from 0.54 (95% CI 0.45 to 0.65) under the primary definition to 0.48 (95% CI 0.39 to 0.60) under the stricter definition. Similarly, the probability of benign MS associated with high fish consumption increased from 1.44 (95% CI 1.28 to 1.64) to 1.51 (95% CI 1.31 to 1.73) (online supplemental etable 3). In contrast, using a broader definition where benign MS was characterised as an EDSS score of 3 or less after 10 years, the associations were slightly attenuated but remained consistent.

## Discussion

Our study contributes novel insights by examining the influence of lifestyle habits on MS progression within the context of benign and non-benign MS. The odds of having benign MS were notably lower among those with a history of IM, higher BMI in young adulthood or low fish consumption. Conversely, frequent fish consumption increased the likelihood of benign MS. Our findings underscore the potential role of lifestyle modifications in influencing disease outcomes.

Our finding that past IM was associated with a higher risk of non-benign MS suggests that this aspect of the Epstein-Barr virus plays a significant role not only in the risk of MS^11 12^ but also in its course. Further investigation is needed to explore underlying potential mechanisms, possibly related to immune perturbations and/or immunogenetic factors. Understanding the association between IM and non-benign MS has several clinical implications. Patients with a history of IM might benefit from closer monitoring for signs of MS progression. Early identification of individuals at higher risk for non-benign MS could lead to more proactive management strategies.

Our study also examined factors associated with the risk of transitioning from benign to non-benign MS after 15 years of disease duration. We found that older age at disease onset, a history of IM, obesity in young adulthood and low fish consumption were associated with an increased risk of transitioning to non-benign MS. These findings suggest that these factors are potential contributors to disease progression in MS beyond initial disease stabilisation.

We observed no significant associations between smoking and low sun exposure at disease onset and the likelihood of having benign MS. It is important to note that this lack of association does not imply that smoking and low sun exposure do not negatively impact disease progression. The potentially harmful effects of these factors on MS progression^13^,^17^ could be obscured in our study by the variability in these behaviours over the course of the disease, bearing the long follow-up in mind.

The significant associations between lifestyle factors and benign MS have important clinical implications. Encouraging smoking cessation,^13 14^ maintaining a healthy weight^18^ and consuming a diet rich in fish may mitigate the risk of disease progression and enhance the overall quality of life for patientsMS.

Our study included a substantial number of patients with MS followed over a considerable duration, using data from the Swedish MS registry^7^ which provided comprehensive and reliable follow-up information. The prevalence of benign MS in our study might have been overestimated, as GEMS used prevalent cases. The most disabled patients might have been excluded due to cognitive impairment or severe disability, leading to a potential selection and over-representation of less severely affected individuals in our analysis. However, when we analysed patients from the incident and prevalent studies separately, the findings remained consistent, indicating that the observed associations are not significantly influenced by the potential selection bias in GEMS. Furthermore, the proportion of patients classified as benign MS in our study is within the expected range.^1^ The associations between lifestyle factors and the probability of a benign disease course remained significant across different definitions of benign MS, indicating the robustness of our findings.

Information regarding lifestyle factors at MS onset relied on retrospective self-reporting which may be subject to recall bias. Despite efforts to use standardised measures, misclassification of exposures could attenuate observed associations. The definition of benign MS relying exclusively on EDSS scores may not capture all dimensions of disease impact, such as cognitive function or more subtle physical impairments. Nonetheless, recent research investigating clinical and genetic factors associated with benign MS has found that patients categorised as benign tend to experience favourable progression across multiple domains of disease, encompassing cognitive function and overall quality of life.^3 4^ While this study identified strong associations between lifestyle factors and MS progression, establishing causality remains challenging within observational studies.

While patients included in our study were diagnosed using the 2005 and 2010 McDonald criteria, this distinction is less critical given that the definition of benign MS relies on the long-term disease course rather than the timing of diagnosis. The 2010 revisions, which enabled earlier diagnosis through simplified MRI criteria, may have influenced the timing of diagnosis for some patients. However, since our analysis is anchored in disease onset, the effect of these diagnostic criteria changes is likely minimal. Theoretically, association could result from reverse causation if patients who later developed non-benign MS experienced early undiagnosed symptoms of MS and consequently changed their lifestyle habits before the disease onset more significantly than non-benign patients with MS. However, past IM and obesity in young adulthood are likely to precede MS onset by many years and dietary habits such as fish consumption are also generally established early in life. Furthermore, it is not likely that patients would reduce their fish intake due to feeling unwell and it is reasonable to infer that these associations reflect potential risk factors rather than the consequences of the disease. To ensure consistency, we used fish consumption at the time of inclusion for both cohorts. Notably, in GEMS, high fish consumption was associated with a higher probability of benign MS both at age 20 and at the time of inclusion, suggesting that dietary habits were relatively stable and supporting the robustness of our findings despite this variation.

Treatment was found to be associated with non-benign MS, likely reflecting the necessity for earlier and more intensive therapeutic interventions in patients with more aggressive disease courses. While this association highlights the potential for confounding by the indication our stratified analysis across different treatment regimens showed consistent findings, supporting the independent influence of lifestyle factors on disease progression.

In conclusion, the probability of a benign disease course in MS was associated with a history of IM and modifiable lifestyle factors. Our findings underscore the potential for targeted interventions to modify disease course and improve outcomes in MS.

## Supplementary material

10.1136/jnnp-2024-335464 online supplemental file 1

## Data Availability

Data are available upon reasonable request.
