# Supplementary material for: Lifestyle factors associated with benign multiple sclerosis
Source: J Neurol Neurosurg Psychiatry. 2025 Feb 13;96(10):e335464. doi: 10.1136/jnnp-2024-335464 (PMC12505036; doi:10.1136/jnnp-2024-335464)
Supplement: online supplemental file 1 [file jnnp-96-10-s001.docx]

**Supplement 1. Included questions from the EIMS and GEMS questionnaires.**

**Ancestry** (EIMS and GEMS)

Were you born in Sweden? No Yes

If “no”, in which country were you born?

What year did you come to Sweden?

What year were your parents born? Mother, year:

Father, year:

Have either of your parents immigrated to Sweden? No Yes

If “yes”, from which country?

Mother, country:

**Educational level** (EIMS and GEMS)

Which of the following schooling/education have you completed? Several alternatives may be chosen.

1. Elementary school, comprehensive school, or the equivalent.

2. Junior secondary school

3. Single-sex school

4. Trade school

5. Vocational school

6. Upper secondary school, high school, grammar school – theoretical programme

7. Upper secondary school, high school, grammar school – practical programme

8. Folk high-school, number of terms:

If applicable, certificate/diploma:

9. University or college, number of terms:

If applicable, degree:

10. Other long-term education, number of terms:

Type of education:

**Infectious diseases that usually occur during childhood or youth** (EIMS and GEMS)

Have you had glandular fever (mononucleosis)?

If “Yes”, at what age do you think you had the disease?

**Smoking habits** (EIMS and GEMS)

Do you smoke?

1. If you do not smoke, and have never smoked, put a cross in the box and proceed to the section “Do you take snuff?” on page 7.

2. If you smoke regularly, or have smoked regularly, put a cross in the box.
Below, specify time period and amount.

Try to specify the average amount. Nb! The number is specified per day.

From age To age Number of cigarettes per day
 Number of cigars/cheroots per day Number of pipe fills per day

3. If you smoke non-regularly, for example at parties, or have smoked non-regularly, put a cross in the box. Below, specify time period and amount.

Try to specify the average amount. Nb! The number is specified per week.

From age To age Number of cigarettes per week
 Number of cigars/cheroots per week Number of pipe fills per week

**Body mass index** (EIMS and GEMS)

1. What is your current weight? kg
(If you are pregnant, please specify your weight at the time prior to the pregnancy)

2. What was your approximate weight when you were 20 years old? kg

3. How tall are you? cm

**Fish consumption habits** (EIMS)

**Specify how often, on average, you have eaten these classes of fish during the last five years.**

1. Fatty fish (i.e. herring/mackerel/tuna-fish/salmon/trout)

Never/seldom 1-3 times/month Every week Daily

**Fish consumption habits** (GEMS)

How often, on average, do you eat fatty fish (for example herring, mackerel, tuna fish, salmon, whitefish, trout)?

1. Never
2. 1-3 times/month
3. Every week
4. Daily

How often, on average, did you eat fatty fish at age 20 (for example herring, mackerel, tuna-fish, salmon, whitefish, trout)?

1. Never
2. 1-3 times/month
3. Every week
4. Daily

**Sun exposure habits** (EIMS)

1. How often, during the last 5 years, have you used a sun-bed?

Never A few times a year Once a month Once a week

2. How often, during the last 5 years, have you visited a country that is sunnier than Sweden?

Never More seldom Once a year More than once a year

3. If the weather is sunny, how often do you usually sunbathe?

Never A few times a month A few times a week Daily

**Sun exposure habits** (GEMS)

Choose one alternative for each period of time below up to your current age. Try to state an average.

How often, on average, have you visited a country that is sunnier than Sweden?

Age
0-9 years 1. Never
10-19 years 2. Less than once a year
20-29 years 3. Once a year
30-39 years 4. Several times a year
40-49 years 5. Not applicable, lived abroad
50 years -

How often, on average, have you sunbathed when the weather was sunny?

Age 1. Never
15-19 years 2. A couple of times per month
20-29 years 3. A couple of times per week
30-39 years 4. Daily
40-49 years
50 years -

How often, on average, have you used a sunbed?

Age 1. Never
15-19 years 2. A few times a year
20-29 years 3. Once a month
30-39 years 4. Once a week
40-49 years
50 years –

eTable 1. OR with 95% CI of benign MS. Patients of Nordic ancestry.

| Characteristic | | Exp BMS/non-BMS | OR (95% CI)^1^ | OR (95% CI)^2^ |
| --- | --- | --- | --- | --- |
| Age at disease onset | <30 years 30-40 years >40 years | 1088/1556 575/1147 209/965 | 1.0 (reference) 0.72 (0.63-0.81) 0.31 (0.26-0.37) | 1.0 (reference) 0.67 (0.59-0.77) 0.23 (0.19-0.28) |
| Sex | Female Male | 1368/2730 504/938 | 1.0 (reference) 1.07 (0.95-1.22) | 1.0 (reference) 1.18 (1.03-1.36) |
| University exam | No  Yes | 1555/3002 317/666 | 1.0 (reference) 0.92 (0.79-1.07) | 1.0 (reference) 0.92 (0.79-1.08) |
| Infectious mononucleosis | No  Yes | 1701/3090 171/578 | 1.0 (reference) 0.54 (0.45-0.64) | 1.0 (reference) 0.51 (0.42-0.61) |
| Smoking | No  Yes | 889/1728 983/1940 | 1.0 (reference) 0.99 (0.88-1.10) | 1.0 (reference) 1.03 (0.91-1.18) |
| Adolescent BMI | < 25 kg/m^2^ Overweight Obesity | 1703/3134 132/391 37/143 | 1.0 (reference) 0.62 (0.51-0.77) 0.48 (0.33-0.69) | 1.0 (reference) 0.65 (0.59-0.88) 0.48 (0.33-0.71) |
| Fish consumption | Never or seldom 1-3 times/month  Weekly | 161/449 984/2097 683/1028 | 0.76 (0.63-0.93)  1.0 (reference) 1.42 (1.25-1.60) | 0.72 (0.59-0.89)  1.0 (reference) 1.48 (1.30-1.70) |
| Sun exposure | Median or higher Below median | 1285/2502 587/1166 | 1.0 (reference) 0.98 (0.87-1.11) | 1.0 (reference) 0.98 (0.86-1.12) |

^1^crude model; ^2^multivariate model; BMS=benign multiple sclerosis. The multi-adjusted models account for all other exposures, as well as age at disease onset, sex, ancestry, and treatment use during the first 15 years.

eTable 2. OR with 95% CI of benign MS, by treatment regimen.

| Untreated or low-efficacy treatment (n=3868) | | | | |
| --- | --- | --- | --- | --- |
| Characteristic | | Exp BMS/non-BMS | OR (95% CI)^1^ | OR (95% CI)^2^ |
| Age at disease onset | <30 years 30-40 years >40 years | 875/790 512/721 195/741 | 1.0 (reference) 0.64 (0.55-0.74) 0.24 (0.20-0.29) | 1.0 (reference) 0.67 (0.57-0.78) 0.23 (0.19-0.28) |
| Sex | Female Male | 1154/1695 428/557 | 1.0 (reference) 1.13 (0.98-1.31) | 1.0 (reference) 1.19 (1.01-1.39) |
| Ancestry | Nordic Non-Nordic | 1467/1963 115/289 | 1.0 (reference) 0.53 (0.43-0.67) | 1.0 (reference) 0.53 (0.42-0.68) |
| University exam | No  Yes | 1312/1845 270/407 | 1.0 (reference) 0.93 (0.79-1.11) | 1.0 (reference) 0.92 (0.77-1.10) |
| Infectious mononucleosis | No  Yes | 1446/1955 136/297 | 1.0 (reference) 0.62 (0.50-0.77) | 1.0 (reference) 0.53 (0.42-0.66) |
| Smoking | No  Yes | 733/975 849/1277 | 1.0 (reference) 0.88 (0.78-1.00) | 1.0 (reference) 1.00 (0.87-1.15) |
| Adolescent BMI | < 25 kg/m^2^ Overweight Obesity | 1440/1963 109/213 33/76 | 1.0 (reference) 0.70 (0.55-0.89) 0.59 (0.39-0.90) | 1.0 (reference) 0.66 (0.52-0.86) 0.54 (0.35-0.84) |
| Fish consumption | Never or seldom 1-3 times/month  Weekly | 139/268 816/1218 594/703 | 0.78 (0.63-0.98)  1.0 (reference) 1.28 (1.11-1.47) | 0.78 (0.61-0.98)  1.0 (reference) 1.38 (1.19-1.60) |
| Sun exposure | Median or higher Below median | 1071/1575 511/677 | 1.0 (reference) 1.10 (0.96-1.25) | 1.0 (reference) 1.06 (0.96-1.21) |
| High-efficacy treatment (n=2489) | | | | |
| Characteristic | | Exp BMS/non-BMS | OR (95% CI)^1^ | OR (95% CI)^2^ |
| Age at disease onset | <30 years 30-40 years >40 years | 314/1080 118/615 26/336 | 1.0 (reference) 0.74 (0.66-0.84) 0.32 (0.27-0.38) | 1.0 (reference) 0.64 (0.50-0.81) 0.23 (0.15-0.35) |
| Sex | Female Male | 333/1464 125/567 | 1.0 (reference) 1.06 (0.93-1.28) | 1.0 (reference) 1.03 (0.82-1.30) |
| Ancestry | Nordic Non-Nordic | 405/1705 53/326 | 1.0 (reference) 0.55 (0.45-0.64) | 1.0 (reference) 0.61 (0.45-0.84) |
| University exam | No  Yes | 386/1648 72/383 | 1.0 (reference) 0.89 (0.78-1.02) | 1.0 (reference) 0.79 (0.60-1.05) |
| Infectious mononucleosis | No  Yes | 404/1675 54/356 | 1.0 (reference) 0.57 (0.48-0.67) | 1.0 (reference) 0.57 (0.42-0.78) |
| Smoking | No  Yes | 236/1036 222/995 | 1.0 (reference) 0.97 (0.88-1.08) | 1.0 (reference) 1.09 (0.89-1.27) |
| Adolescent BMI | < 25 kg/m^2^ Overweight Obesity | 406/1693 46/250 6/89 | 1.0 (reference) 0.67 (0.55-0.81) 0.47 (0.33-0.67) | 1.0 (reference) 0.76 (0.54-1.06) 0.26 (0.11-0.61) |
| Fish consumption | Never or seldom 1-3 times/month  Weekly | 43/278 264/1221 135/478 | 0.76 (0.63-0.90)  1.0 (reference) 1.40 (1.25-1.57) | 0.68 (0.48-0.97)  1.0 (reference) 1.34 (1.05-1.69) |
| Sun exposure | Median or higher Below median | 338/1365 120/666 | 1.0 (reference) 0.98 (0.88-1.10) | 1.0 (reference) 0.76 (0.60-0.97) |

^1^crude model; ^2^multivariate model; BMS=benign multiple sclerosis. The multi-adjusted models account for all other exposures, as well as age at disease onset, sex, ancestry, and treatment use during the first 15 years.

eTable 3. OR with 95% CI of benign MS.

| Characteristic | | Exp BMS/non-BMS | OR (95% CI)^1^ | OR (95% CI)^2^ |
| --- | --- | --- | --- | --- |
| Age at disease onset | <30 years 30-40 years >40 years | 1189/1870 630/1336 221/1077 | 1.0 (reference) 0.71 (0.63-0.81) 0.32 (0.27-0.38) | 1.0 (reference) 0.65 (0.56-0.74) 0.22 (0.18-0.27) |
| Sex | Female Male | 1487/3159 553/1124 | 1.0 (reference) 1.15 (1.01-1.31) | 1.0 (reference) 1.30 (1.13-1.49) |
| Ancestry | Nordic Non-Nordic | 1872/3668 168/615 | 1.0 (reference) 0.51 (0.42-0.63) | 1.0 (reference) 0.50 (0.40-0.62) |
| University exam | No  Yes | 1698/3493 342/790 | 1.0 (reference) 0.92 (0.79-1.07) | 1.0 (reference) 0.95 (0.81-1.12) |
| Infectious mononucleosis | No  Yes | 1850/3630 190/653 | 1.0 (reference) 0.51 (0.42-0.62) | 1.0 (reference) 0.48 (0.39-0.60) |
| Smoking | No  Yes | 969/2011 1071/2272 | 1.0 (reference) 1.00 (0.89-1.12) | 1.0 (reference) 1.04 (0.92-1.19) |
| Adolescent BMI | < 25 kg/m^2^ Overweight Obesity | 1846/3655 155/463 39/165 | 1.0 (reference) 0.56 (0.45-0.70) 0.41 (0.27-0.63) | 1.0 (reference) 0.57 (0.45-0.73) 0.41 (0.26-0.63) |
| Fish consumption | Never or seldom 1-3 times/month  Weekly | 182/546 1080/2439 729/1181 | 0.82 (0.68-0.99)  1.0 (reference) 1.45 (1.28-1.64) | 0.78 (0.63-0.97)  1.0 (reference) 1.51 (1.31-1.73) |
| Sun exposure | Median or higher Below median | 1409/2940 631/1343 | 1.0 (reference) 1.05 (0.93-1.19) | 1.0 (reference) 1.04 (0.92-1.21) |

^1^crude model; ^2^multivariate model; BMS=benign multiple sclerosis (EDSS less than 3 after 15 years of disease duration). The multi-adjusted models account for all other exposures, as well as age at disease onset, sex, ancestry, and treatment use during the first 15 years.
